# Classification of Alzheimer's and MCI Patients from Semantically Parcelled PET Images: A Comparison between AV45 and FDG-PET

**DOI:** 10.1155/2018/1247430

**Published:** 2018-03-15

**Authors:** Seyed Hossein Nozadi, Samuel Kadoury, The Alzheimer's Disease Neuroimaging Initiative

**Affiliations:** ^1^Department of Computer and Software Engineering, Polytechnique Montreal, P.O. Box 6079, Downtown Station, Montreal, QC, Canada H3C 3A7; ^2^Center for Imaging of Neurodegenerative Disease, San Francisco VA Medical Center, University of California, San Francisco, CA, USA

## Abstract

Early identification of dementia in the early or late stages of mild cognitive impairment (MCI) is crucial for a timely diagnosis and slowing down the progression of Alzheimer's disease (AD). Positron emission tomography (PET) is considered a highly powerful diagnostic biomarker, but few approaches investigated the efficacy of focusing on localized PET-active areas for classification purposes. In this work, we propose a pipeline using learned features from semantically labelled PET images to perform group classification. A deformable multimodal PET-MRI registration method is employed to fuse an annotated MNI template to each patient-specific PET scan, generating a fully labelled volume from which 10 common regions of interest used for AD diagnosis are extracted. The method was evaluated on 660 subjects from the ADNI database, yielding a classification accuracy of 91.2% for AD versus NC when using random forests combining features from cross-sectional and follow-up exams. A considerable improvement in the early versus late MCI classification accuracy was achieved using FDG-PET compared to the AV-45 compound, yielding a 72.5% rate. The pipeline demonstrates the potential of exploiting longitudinal multiregion PET features to improve cognitive assessment.

## 1. Introduction

Alzheimer's disease (AD) is one of the most common types of neurodegenerative disorders in the aging population [1]. A recent research by the Alzheimer's association reports that AD is the sixth-leading cause of death in the United States and is rising every year considering its proportion in the causes of death [2]. The first signs of AD will typically include forgetfulness and will progress by affecting various functions such as language, motor skills, and memory [3]. However, a slight but noticeable and measurable decline in cognitive abilities, including memory and reasoning abilities, can be associated with mild cognitive impairment (MCI). An individual diagnosed with MCI could be at risk of later developing Alzheimer's, or can be due to age-related memory decline, thus highlighting the importance of early diagnosis of the disease. Still, clinical and neuroimaging studies have demonstrated differences between MCI and normal controls (NC) [4, 5]. Patients diagnosed with MCI can be stratified between early MCI (EMCI) and late MCI (LMCI) [6].

There is no definitive cure for AD, whereas active research areas seek treatments which are more effective for early MCI in order to slow down the progression of the disease. This implies a great urgency to develop sensitive biomarkers to detect and monitor early brain changes. The ability to diagnose and classify AD and MCI at an early stage allows clinicians to take more informed decisions at later stages for clinical intervention and treatment planning, thus having a great impact on reducing the cost of longtime care [7]. From a neuroimaging perspective, positron emission tomography (PET) of fluorodeoxyglucose (FDG) for cerebral glucose metabolism and *β* amyloid (also known as AV45 or florbetapir) can provide complementary information for the diagnosis of AD [8–11].

The classification between AD, MCI at its two levels (EMCI and LMCI), and NC using PET imaging is a topic that has been studied by previous groups. Feature-based methods to classify various cognitive states have used several characteristics such as brain volume [12], ratio of voxel intensities represented by standardized uptake values (SUV) [13, 14], or mean voxel intensities (VI) [15] which were extracted from the PET volume. Voxel intensities in particular have been shown to be one of the most discriminant features for classification purposes [16, 17]. For instance, Smailagic et al. used VI to reduce the number of voxels by excluding lower intensity values from the PET image and simplify the classification process [14]. Gray et al. differentiated AD and NC with a classification accuracy of 88%, whereas nonconvertible MCI and NC yielded a 65% accuracy [9]. The authors used FDG-PET images from the Alzheimer's Disease Neuroimaging Initiative (ADNI). Their method proposed to classify AD versus nonconvertible MCI and convertible MCI to test regional feature sets based on signal intensities. In works by López et al. [18], feature extraction was performed using principal component analysis (PCA) on whole-brain images, achieving accuracies up to 89%. In a work by Rodrigues and Silveira using the same longitudinal database, the best classification accuracy was obtained by combining two timepoints (baseline and 12-month follow-up) (93% for NC versus AD) [19]. In another study, Aidos et al. used VI in some ROIs which were manually segmented by experts [20]. By using an automated feature selection method to classify the subjects, they used mutual information to rank the features and identify the highest values to reduce the number of features. The optimal classification rates were achieved using support vector machines (SVMs) or* k*-nearest neighbor (KNN) by using selected regions for MCI, with rates ranging between 65% and 79%. An accuracy of 85% for AD versus NC was also obtained using the same method. However, all these previous studies exploited a specific glucose molecule (primarily FDG), without comparing either FDG or AV45 for classification purposes, and did not include an automatic parcellation of the specific cortical regions particularly prone to amyloid accumulation to achieve improved classification rates. A recent review summarizes feature-based methods [21].

In this work, we evaluate the diagnostic potential of PET images from automatically extracted cortical and subcortical regions, while comparing the diagnostic efficacy of FDG-PET with that of AV45-PET. In the first approach (hereafter called multiregion approach), we developed a method to segment the ROIs and extract the features while in the second we used a similar method but for the entire brain volume (hereafter called whole-brain approach). Due to the subtle differences in imaging, EMCI and LMCI are a particularly challenging problem for classification, especially using amyloid imaging such as PET. Generally, most of the studies compared AD, MCI (single class), and NC [9, 12, 16, 22]. Therefore in this study, datasets were grouped into four classes (AD, EMCI, LMCI, and NC) and performed predictions using PET images only. Each subject had two PET scans at two timepoints (baseline and after approximately 12 months), FDG-PET and AV45-PET images, which were used solely for comparative purposes. We hypothesized that longitudinal PET images provided additional information to achieve a high level of discrimination between EMCI and NC, LMCI and NC, or EMCI and LMCI. The main objective is to develop a method to select the optimal features using PET images alone to classify AD, EMCI, LMCI, and NC. The framework is evaluated with a large sample size of 660 subjects, in contrast to previous works which used a more limited number of subjects (under 250) [9, 19, 20]. Furthermore, while previous methods were proposed to predict the conversion [22, 23], they were designed for whole-brain processing and did not focus on particular regions prone to amyloid accumulation.

We propose to classify subjects by either using only the baseline PET images or combining a 12-month follow-up examination (the second scan of ADNI subjects was acquired between 6 and 18 months after baseline; this period of time is not the same for all subjects which gives an approximate 12-month period) (hereafter called second-visit). First, following a preprocessing step to normalize the input images, a nonrigid registration step between magnetic resonance images (MRI) and PET images is applied, followed by a nonrigid transformation to the MNI152-space which was annotated by the automated anatomical labelling (AAL) brain atlas. To extract features from each area, we used this AAL labelling method and selected 10 regions of interest (ROIs) in each subject. The second-visit images underwent the same process to extract the features and combined with the baseline for the multiregion approach for AV45-PET and FDG-PET images individually. The whole-brain approach used voxel-wise features from the entire volume for the same images and timepoints. For classification purposes, we used as basis of comparison both linear and RBF SVMs, SVM using PCA reduced features, and random forests (RF). The paper is structured as follows.  Section 2 presents the materials and the methodology for the classification pipeline.  Section 3 presents the experimental results, followed by a discussion.  Section 4 concludes the paper.

## 2. Materials and Methods

**Figure 1:**
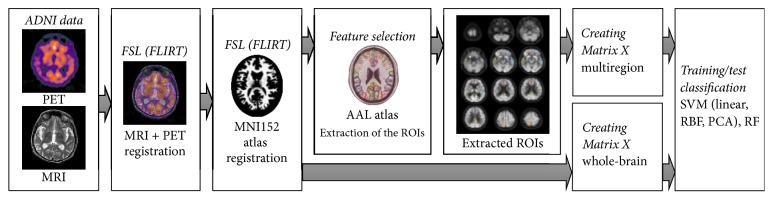
Overview of the proposed method.

We propose a pipeline including PET and MRI registration, atlas annotation, anatomical segmentation, feature selection, and classification to automatically process input PET images for AD/LMCI/EMCI/NC classification, as shown in Figure 1. The following sections describe each component of the pipeline.

### 2.1. Data Selection

The data used in this work was from the ADNI database (http://www.loni.ucla.edu/ADNI). ADNI is an ongoing longitudinal, multicenter study launched in 2003, designed to develop clinical, imaging, genetic, and biochemical biomarkers for the early detection and tracking of AD. It includes approximately 1800 subjects recruited from over 50 sites across the US and Canada in three databases (ADNI, ADNI GO, and ADNI 2) [24] (http://adni.loni.usc.edu/). Among all subjects which are available for download, this study collected resting-state FDG-PET brain volumes acquired at two timepoints: baseline and second-visit (for comparison purposes). The total number of subjects was 1046 patients; 386 patients were excluded because they did not have the complete PET series and/or corresponding MRI images at the same time. A final set of 660 subjects with PET and MRI images as well as MMSE scores at both timepoints was selected, which amounted to 99 AD, 164 EMCI, 189 LMCI, and 208 NC.  Table 1 presents the characteristics of the patient cohorts.

### 2.2. PET and MRI Datasets

#### 2.2.1. PET Protocol

FDG-PET is a metabolic neuroimaging modality frequently used in AD, producing a distribution map of glucose uptake. The reduction of glucose uptake in the temporal and parietal areas is often seen as an indication of AD [4]. Foster et al. demonstrated that FDG-PET improves the accuracy of differentiating AD from frontotemporal dementia, especially when the symptoms and clinical tests are equivocal [25]. However other types of tracers exist such as florbetapir which helps to image amyloid accumulation in the brain.

In this study, PET images were preprocessed in four steps. These steps include (1) coregistration between sequences to reduce the effect of patient motion and converting it into DICOM format, (2) mean averaging of the 6 five-minute frames of the* coregistered dynamic* image set, (3) reorientation of the output images into a standard space with 160 × 160 × 96-voxel image grid of 1.5 mm cubic voxels, and (4) acquiring a uniform image with isotropic resolution of 8 mm full width at half maximum [24] (http://adni.loni.usc.edu/). Scans were further processed with range normalization to provide uniform datasets. An intensity normalization was required in order to perform direct images comparisons between different subjects. The intensity of the PET images was normalized based on the maximum value *I*_max_, determined from the mean of voxel intensities over the value *T*_max_. Here, *T*_max_ was determined by the intensity value of the 10th histogram bin (using a total of 50 bins). These steps help to discard all low-valued intensity voxels outside the brain and avoid image saturation.

#### 2.2.2. MRI Protocol

The MR images were acquired on either Philips, GE, and Siemens scanners from across all participating sites. Since the acquisition protocols were different in each scanner, an image normalization step was provided by ADNI. Corrections included geometry distortion, calibration, and reduction of intensity of nonuniformity. More detailed information is available on the ADNI website (http://adni.loni.usc.edu/). These corrections were applied to each MPRAGE volume following the image preprocessing steps. We used T1-weighted images which were selected and reviewed for quality and correction in terms of data format and/or alignment. MR images were used only to find the subcortical anatomical structures to increase the labelling accuracy of the ROIs. Finally, the MRI with 176 × 256 × 256 resolution with 1 mm spacing was collected with the purpose of increasing the accuracy of registration and labelling.

### 2.3. Region Extraction from PET

#### 2.3.1. PET-MRI Registration

Proposing an accurate multimodal registration step between PET and MRI is crucial in the proposed pipeline as it dictates the reliability of PET segmentation and subsequently provides better results for the ROI extraction. The first step was to register the PET image and its corresponding T1-w MRI. In our pipeline, skull stripping was not necessary as images were already preprocessed, so, by reducing the total processing steps from the original images, high-dimensional information of signal intensities was preserved as much as possible for the feature-learning step. Choosing the appropriate registration parameters, similarity function, and transformation model becomes particularly important. While available tools such as FLIRT were previously validated in other studies [26, 27], the registration accuracy between PET and MRI using a subset of patients from ADNI was performed in this study, comparing both the SPM and FSL tools (FLIRT toolkit) and measuring the anatomical landmark residual errors. These landmarks were identified on the images by an experienced neuroradiologist, and the Euclidean distance between landmarks identified on the MRI with transformed points from the PET image was calculated. The evaluation was performed by cross-validation on a separate subset of 20 subjects with FDG-PET and MRI from the ADNI database, including 10 AD and 10 NC patients.

We compared the registrations between PET and MRI using four different scenarios, based on transformation model (rigid versus affine) and with different cost functions (mutual information versus correlation ratio). Because there is no common gold standard for registration between PET and MRI, we used the same method as similar works, using a previously validated PET simulation based on corresponding MRI [28, 29].

The simulated PET images were generated by a series of steps which includes scalp editing and segmenting the MRI images into GM, WM, and CSF, which is done using the Seg3D tool version 1.10.0 (Scientific Computing and Imaging Institute, University of Utah). The voxel intensities of GM, WM, and CSF were modified and a rigid body or affine transformation with resampling to the PET voxel size was applied. Finally, an 8 mm FWHM Gaussian filter was used to smooth the images and remove residual noise. This process was repeated for 16 iterations on each image.

**Figure 2:**
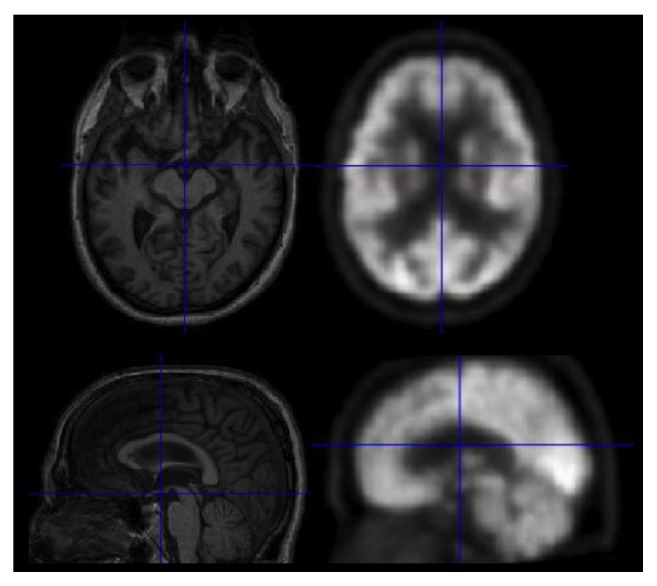
Selected regions of interest identified on PET images for cognitive classification tasks.

Results are presented in Table 2.  Figure 2 shows an example of the resulting registration between PET and MRI using FLIRT. These quantitative comparisons show that the FLIRT tool is able to achieve improved accuracy in terms of registration error, using an affine transformation with correlation ratio used as the similarity measure for all 660 subjects in two timepoints as it provided the best intermodality registration accuracy.

#### 2.3.2. Atlas Registration

Once the PET and MRI images were registered for each patient, the native patient MRI was registered to a common coordinate space for multiregion extraction (MNI152 template) to facilitate the localization of the analyzed regions. In order to reduce the significant amount of time and effort required to segment and label the set of PET-active anatomical structures listed in the following section from the 3D MRI brain images of the selected cohort of patients, we used an anatomical correspondence estimation relating the atlas to the target image space, thus increasing the accuracy of the resulting target labelling [30]. For the purpose of anatomical segmentation to extract the ROIs, we used the 2 mm resolution atlas which was adapted for registration accuracy. This labelling technique provides 116 cortical and subcortical labels which are accurate and are perfectly compatible with the MNI152 atlas [31]. Registration with AAL enables segmenting the whole-brain structure and choosing the ROIs. In order to perform voxel-wise analysis, all images were registered to the uniform size of the MNI template. Therefore, after PET and MRI registration, the resulting images were nonrigidly registered once more with MNI152 atlas (2 mm) using the open source software NiftyReg. Thus, we obtained all images with 91 × 109 × 91 resolution, with 2 mm isotropic voxel size.

#### 2.3.3. Extraction of ROIs

**Figure 3:**
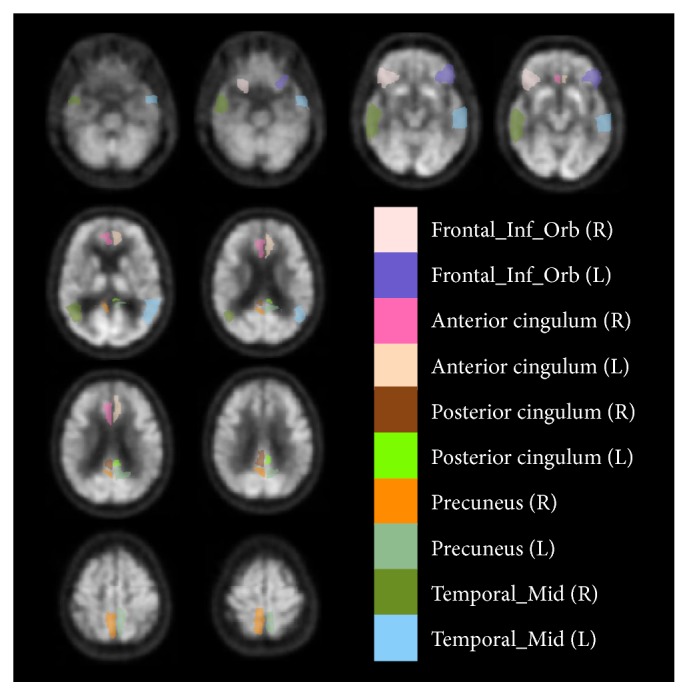
Selected regions of interest identified on PET images for cognitive classification tasks.

FDG-PET or AV45-PET images in dementia show specific regions presenting patterns of abnormality caused by the disease. Therefore from the 116 regions of the brain identified using our registration technique, five regions in particular from each hemisphere were studied for the accumulation of glucose, namely, (1) anterior cingulum (right and left), (2) posterior cingulum (right and left), (3) inferior frontal gyrus/orbitofrontal (right and left), (4) precuneus (right and left), and (5) lateral temporal (right and left). These patterns appear in multiple neurological disorders, including Alzheimer's disease. The main reason to extract these ROIs is to extract metabolic data from specific anatomical areas within the brain volume. These ROIs were previously identified in the literature [32–34] and in the neuroscience field. Previous studies on AD classification did not use the previously mentioned regions which are more likely to be affected by AD [35, 36], concentrating rather on a whole-brain analysis. In order to obtain the appropriate labels for the current study, AAL was used to label each region of the MNI brain space. Our method is based on the parcellation of the MNI152 brain template with the AAL atlas. These ten selected regions were therefore extracted from the normalized PET image by selecting the specific labels after the AAL registration technique and regional extraction in the original image volume space (Figure 3).

Binary masks for each of the 10 ROIs were subsequently created in the automated pipeline before applying it on the processed images to collect SUV voxels. Each voxel from the labelled ROIs obtained from segmentation was used in this process. A single mask combining all 10 separate masks together was then created, thus representing a single feature vector of all regions which had more probability of being affected by AD and specifically had less accumulation of glucose. After applying the mask, we then replaced the mask ROIs labels by the voxel values from the raw data for each subject. Following that, the data and images were ready for feature extraction.

### 2.4. Feature Extraction

Preprocessed subject images each had normalized binary masks representing the different labelled ROIs, from which the corresponding intensity values can be used for subsequent feature extraction. From these voxel intensities values, assuming that the number of voxels differed for each region *k*_*i*_, each subject dataset *S*, which included 10 ROIs, can be defined as follows:

(1) $$\begin{array}{c} S=\left\{\;k_{1},k_{2},k_{3},\ldots ,k_{10}\;\right\}. \end{array}$$

Given *N* = 660 subjects with a vector of voxels associated with each subject, we reshaped the 3D matrix for all subjects, ROIs, and voxels/per ROI, to a single feature vector. However, because the sum of voxels for each region *k*_*i*_ is different for each subject, the size of each vector was normalized based on the maximum number of voxels among all subjects for each ROI. Based on the maximum identified values, we calculated the maximum length of the overall vector such that

(2) ∑i=110 ∑j=1Nmax⁡kij,N=660,X=S1=k1,k2,k3,…,k10,…,S660=k1,k2,k3,…,k10.

**Figure 4:**
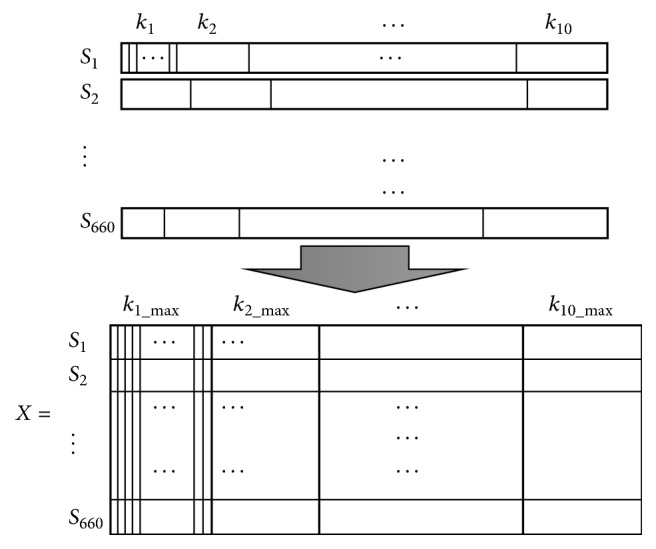
Dimensionality reduction of the feature matrix *X*, containing training subjects from the ADNI dataset, used to train the classifiers.

Using this approach, we created the matrix *X* with vector of the subjects as rows and a maximum number of valued voxels (*k*_max_) as columns (Figure 4).

In the proposed multiregion approach, we used matrix *X* which had a dimensionality of *n* × 660 in order to train the classifiers, with *n* the total number of voxels, whereas, in the whole-brain approach, the size of the feature matrix includes all valued voxels in the image. The objective was to learn the features from the VI in order to discriminate classes. The main difference between the two approaches was the total number of voxels used.

### 2.5. Classification

Once the PET-based features were extracted from the set of selected ROIs located in both cortical and subcortical regions, feature vectors containing mean-centered voxel intensities were created combining each of the 10 ROIs and assembled for all cases. Supervised classification was performed using four different multiclass methods, which included (1) linear SVM on raw voxel intensities, (2) RBF kernel SVM on raw voxel intensities, (3) SVM trained with features extracted using principal component analysis (PCA), and (4) random forests (RF) classifier. Hyperparameters of the RBF kernel were obtained using an exhaustive search grid (described in Section 3), where the parameters were selected based on the maximum in-sample validation accuracy which outperformed polynomial kernels. The tuned hyperparameters were then used to predict the out-of-sample accuracy values on the test set.

## 3. Results and Discussion

### 3.1. Parameter Selection

We then present the methodology to determine the optimal parameter settings of each classifier. In order to train the RBF-SVM classifiers, two hyperparameters need to be determined: the penalty parameter *C* and the kernel width *γ*. Considering the number of features and data for training and testing, a properly tuned RBF kernel was shown in preliminary testing to be superior in terms of accuracy to linear kernels, even though they are simpler to use [37].

Cross-validation was used for tuning the RBF kernel. To evaluate the performance of different classification methods and find the best hyperparameters for SVM classifiers, we used a 10-fold cross-validation strategy. In order to determine the hyperparameters of the SVM-RBF kernel, an exhaustive grid search on the *C* and *γ* parameters was performed based on classification accuracy, where 10 subjects were randomly selected from the dataset for testing, and the remaining unseen subjects (*N* − 10) were used to train the classifiers. This procedure was repeated 1000 times, each time randomly selecting a new set of 10 held-out subjects to test classification performed based on the set of hyperparameters under the performance converged. The maximum in-sample validation accuracy was found at *C*_opt_ = 1 and *γ*_opt_ = 0.1. The tuned hyperparameters were used to predict the out-of-sample accuracy values on the test set. As for the PCA approach, principal components were calculated such that 95% of the group variance was retained. To ensure we did not observe any overfitting in our data, we performed an experiment which progressively increased the PC-subset inclusion from 70% to 99% (with 1% increments) using cross-validation testing and chose the value which offered the best performance before seeing overfitting of the data, where reconstruction values stabilized, yielding a value of 95%. Finally, the number of trees grown in each forest (*t*) and the number of features (*f*) randomly selected at each tree node had to be determined. Based on out-of-bag classification errors to measure stability of training, we found that *t* = 60 and that $f=\sqrt{D}$ for all the experiments, where *D* is the initial dimensionality of the vectors, based on the findings of Liaw and Wiener (2002).

### 3.2. Results

We begin by presenting the results of the multiregion approach, followed by the whole-brain approach, both using FDG-PET images. This is followed by a more detailed analysis of the longitudinal classification experiments, with the combination of two timepoints (baseline and 12-month). Finally we present the comparative results between FDG-PET and AV45-PET.

In this work, we report the results of classification between six different paired classes of cognitive states using four classifiers in two approaches (multiregion and whole-brain). The results of four paired classes (AD versus NC, AD versus EMCI, AD versus LMCI, and EMCI versus LMCI) of cognitive states are presented in Tables 3 and 4.

#### 3.2.1. Multiregion Classification

We first used the proposed methodology to segment and classify the baseline image based on regional signal intensities (ROIs). As shown in Tables 3(a) and 3(b), RF and RBF-SVM demonstrated higher accuracies (over 80%). In Tables 3(c) and 3(d), although values were globally lower, these classifiers still had the highest accuracies (over 65%), with the exception of the SVM classifier which has 70% accuracy in the second visit (Table 3(d)).

#### 3.2.2. Whole-Brain Classification

In comparison to the region-based approach, the whole-brain technique did not perform as well for AD versus NC and for AD versus EMCI as shown in Tables 4(a) and 4(b). On the other hand, the RF classifier for the whole-brain approach demonstrated the highest accuracies for AD versus LMCI and EMCI versus LMCI (81.7% and 72.5%, resp.) compared to the other classifiers which were between 55% and 68.2%. These results also outperform the region-based approach using the baseline scan for these last two pairs.

#### 3.2.3. Combination of Longitudinal Data

**Figure 5:**
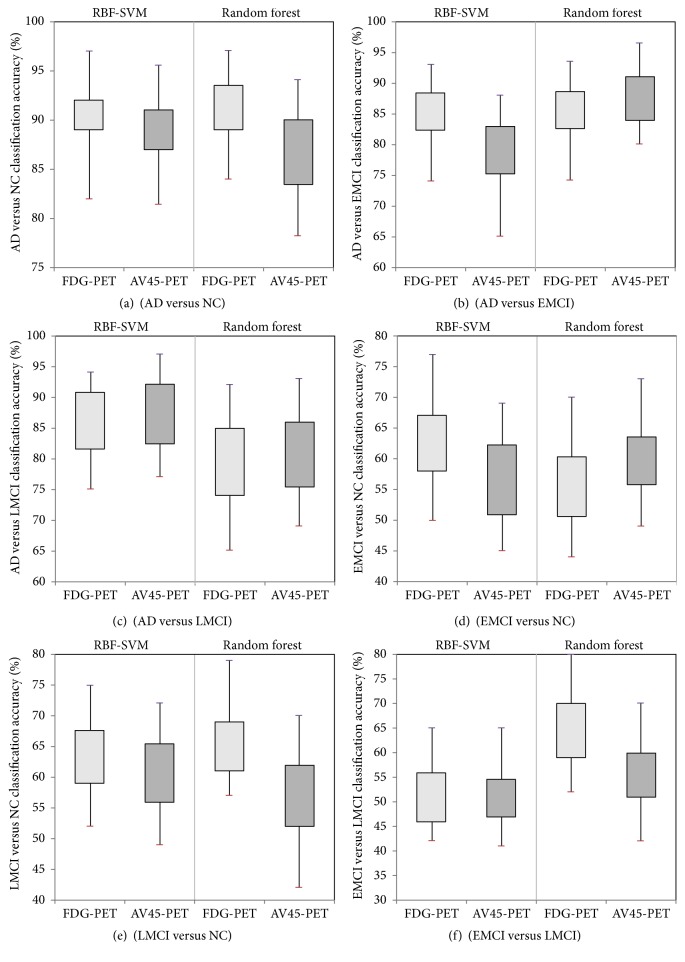
Box-plot figures of classification accuracies using multiregion approach between FDG-PET and AV45-PET, combining baseline and second-visit timepoints for all diagnostic pairs.

The classification results using the combination of two timepoints in the multiregion approach were obtained by combining the second-visit data with the baseline data. Based on the literature and the previous results, we applied RBF-SVM and RF on the combined data. Features matrices therefore combined the extracted information from the multiregion approach from visits 1 and 2, which doubled the size of matrix *X*. The highest classification accuracy was obtained for AD versus NC based on FDG-PET images using RBF-SVM (91.7%) and RF (91.2%) methods. These results are shown in Table 5 and Figure 5 and are higher than baseline and second-visit results taken individually (Table 3).

**Figure 6:**
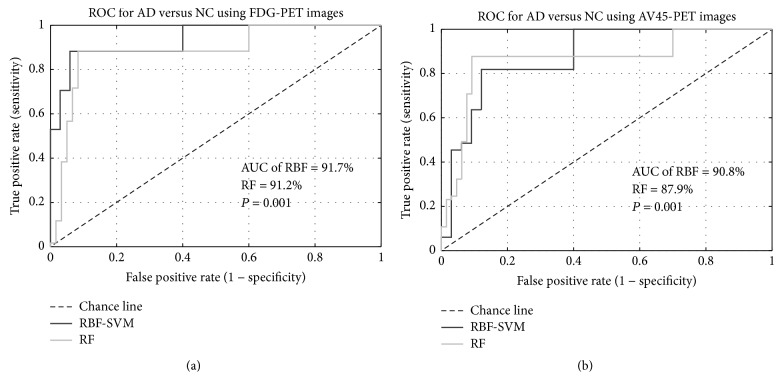
Receiver Operating Characteristic (ROC, sensitivity versus specificity) curves for classification between AD and NC using longitudinal data for (a) FDG-PET images (AD versus NC) and (b) AV45-PET images.

Figure 6(a) shows the Receiver Operating Characteristic (ROC) curves, for the results based on Table 5. Classification accuracies of AD versus NC for FDG-PET in RBF-SVM and RF were similar but still significantly different based on McNemar's test (McNemar, 1947) to determine whether this is a substantial difference.

#### 3.2.4. AV45-PET versus FDG-PET

Finally, we compared the diagnostic accuracy between AV45-PET and FDG-PET images. Table 5 presents the results of the multiregion approach using combined data for AV45-PET images (right columns) compared to the results of FDG-PET images (left columns). These results demonstrate the high accuracy of RBF-SVM and RF classifiers for AD versus NC (90.8% versus 87.9%), AD versus EMCI (80.0% versus 88.0%), and AD versus LMCI (88.9% versus 81.5%). As previously mentioned for the FDG-PET results, higher accuracies were achieved when using the combination of the two timepoints compared to individual timepoint results.

Figure 6(b) illustrates the ROC curves for the classification results based on Table 5. Classification accuracies of AD versus NC for AV45-PET in RBF-SVM and RF were similar but still significantly different (*P* = 0.02).

### 3.3. Discussion

To the best of our knowledge, this is one of the few studies to focus on PET classification for cognitive stage identification in AD, comparing learned features from both AV45-PET and FDG-PET images, while using a multiregional approach based on segmented cortical and subcortical areas. The objective was to assess how a feature-learning approach focused on predefined anatomical regions with known decline in uptake for AD patients can help achieve better accuracy and minimize the errors of an automated classification of different stages of Alzheimer's, especially in the early stages of the disease. The classification results in this work are comparable and, in some cases, better than the performances reported in the literature [9, 16]. Generally, classification accuracy between AD and NC is a typical benchmark to compare different methodology performances. Classification between the different stages of MCI, namely, EMCI and LMCI, is a more challenging issue in this field due to subtle differences which are not noticeable from the human perception. In fact, most publications discriminate MCI (combining both late and early stages) and AD, with fewer attempts on EMCI or LMCI [12, 16, 19]. Considering the importance of having the results of early and late stages of MCI versus NC, we conducted additional experiments to demonstrate that the models can indeed discriminate between early and late stages of MCI.

We demonstrated that AV45-PET images as well as FDG-PET images offer relevant and discriminative features to yield classification results which are comparable to models using imaging and nonimaging data [15, 16]. Results were slightly better with FDG-PET due to increased spatial resolution in the image, which helps to delineate the localized structures affected by AD. The main motivation of this study was to directly compare the classification efficiencies of FDG versus AV45, which was done by training separate classifiers and evaluating the performance between pairs of cognitive groups. Our goal was to uncover which radiotracer demonstrated the stronger diagnostic accuracy and come to a recommendation of the tracer to use for AD. While both FDG and AV45 images were indeed available in ADNI for research purposes, typically in a clinical examination, FDG and AV45 will not be acquired during the same session, but rather one or the other. In a subsequent study, we will investigate the combination of both modalities, which, by combining features from both images which tend to highlight different anatomical regions affected by AD, could improve the overall classification accuracy.

Regarding the atlas registration step, it was crucial to choose an atlas offering all segments of the brain anatomy which would be included in our predefined ROIs. To achieve this objective, we explored a number of different atlases, including the Harvard-oxford cortical and subcortical structural atlas [38], the Talairach atlas [35, 39], the MNI152 structural atlas [40], and the automated anatomical labelling (AAL) atlas [31]. Among all four atlases, only AAL included all 10 ROIs of interest from the segmented 116 cortical and subcortical regions, which were preidentified in the literature as prone to amyloid accumulation. Therefore, the MNI152 atlas combined with AAL was selected for the purposes of our pipeline.

The results obtained in this study using the ADNI-PET images were similar to previous works [9, 20], which also used VI for feature-learning. This confirms the high diagnostic power of uptake values in discriminating between different cognitive stages, particularly by integrating a progression component using time series features to the analysis.

To achieve these results, multimodal registration played another key role to properly align both PET and MRI. The FLIRT tool was able to register intra- and intermodal brain images without extracting or segmenting the whole volume of the brain, which is convenient for processing large cohorts of patients with the proposed pipeline, by using automated registration for all subjects.  Table 1 displays the MMSE for both groups of AD and NC for the two timepoints. It confirms a significant progression of the disease from baseline to the second-visit exam as observed in the results of the two approaches.

Results using PCA-SVM see little changes in most diagnostic pairs between baseline and second visit, compared to RBF-SVM or RF classifiers. This indicates that the additional PET features provided in the longitudinal scans are nonlinear features better detected by kernel- or learning-based methods. From these results, we can also interpret that combining second-visit data provides additional information to discriminate between the classes compared to the baseline data. Finally when comparing both the ROIs and whole-brain approaches, results at each timepoint individually were better with the ROIs method, except when using the linear SVM classifier. The combination between cross-sectional and longitudinal information achieved very good accuracy for AD compared with other classes but as presented in Table 5 for EMCI versus NC, LMCI versus NC, and EMCI versus LMCI, it is not as adequate as with longitudinal data alone.

## 4. Conclusion

In this work, we compared both whole-brain and multicortical region approaches to identify cognitive stages of AD, comparing both FDG-PET and AV45-PET in classification accuracy. We observed that the classification accuracy of AD versus NC was improved using longitudinal images, as well as for other pairs of cognitive classes. Either FDG-PET or AV45-PET enabled discriminating early and late MCI from AD, as well as NC, with a slight improvement using FDG-PET. The methodologies used in this work can contribute to improving the classification accuracy between different stages of AD by using the combination of two timepoints. Our results confirm that we can rely on PET images as a single biomarker, although the inclusion of additional biomarkers can also improve the accuracy of classification. Results were shown to be favorable or better compared to previous studies, especially for the more challenging classification tasks such as NC versus EMCI, AD versus LMCI, or EMCI versus LMCI. Future work will involve combining additional biomarkers such as cortical thickness data, volume, voxel-wise tissue probability, and density of gray matter, in comparison with deep classifiers and other state-of-the-art AD classification approaches. In the context of Alzheimer's disease, the method can improve for the early detection of the disease with promising classification rates based on ground-truth knowledge.

## Figures and Tables

**Table 1:** Participant distribution.

Characteristics	AD	EMCI	LMCI	NC
*n *	99	164	189	208
Mean age (yrs)	75.7 ± 8.2	74.3 ± 8.1	72.8 ± 7.0	76.3 ± 8.4
Gender (M/F)	57/42	94/70	101/88	119/88 + 1 (undefined)
MMSE				
Baseline	22.9 ± 2.2	28.5 ± 1.4	27.6 ± 1.8	29.1 ± 1.2
Second-visit	20.4 ± 4.1	27.7 ± 2.7	24.4 ± 4.9	28.3 ± 2.3

**Table 2:** Configuration of cost function and transformation model (rigid with 6 parameters versus affine with 12 parameters) of registration, comparing SPM and FLIRT tools based on landmark registration errors (LRE).

Configuration	Cost function	Transformation	LRE with SPM (mm)	LRE with FLIRT (mm)
(1)	Mutual information	Rigid body	3.15 ± 1.10	3.02 ± 1.11
(2)	Correlation ratio	Rigid body	2.26 ± 0.92	2.13 ± 0.86
(3)	Mutual information	Affine	2.65 ± 0.97	2.51 ± 0.41
(4)	Correlation ratio	Affine	1.12 ± 0.45	0.98 ± 0.31

**(a):** 

Method	Timepoints	ACC	SEN	SPE	AUC
SVM	1st visit	81.7	83.5	72.8	0.843
	1st + 2nd visit	81.0	83.2	70.8	0.839
PCA	1st visit	85.7	84.3	79.2	0.876
	1st + 2nd visit	87.1	86.1	80.6	0.884
RBF	1st visit	88.2	86.4	85.2	0.894
	1st + 2nd visit	89.3	88.8	85.9	0.900
RF	1st visit	88.2	89.5	86.8	0.901
	1st + 2nd visit	89.7	89.1	87.0	0.903

**(b):** 

Method	Timepoints	ACC	SEN	SPE	AUC
SVM	1st visit	70.0	76.3	59.8	0.745
	1st + 2nd visit	74.0	81.4	62.3	0.767
PCA	1st visit	74.5	77.2	65.9	0.772
	1st + 2nd visit	74.0	79.7	66.0	0.778
RBF	1st visit	76.5	79.1	67.4	0.791
	1st + 2nd visit	81.7	86.5	70.3	0.866
RF	1st visit	82.4	85.2	76.9	0.874
	1st + 2nd visit	82.4	85.3	76.9	0.876

**(c):** 

Method	Timepoints	ACC	SEN	SPE	AUC
SVM	1st visit	60.0	66.5	69.2	0.675
	1st + 2nd visit	63.2	70.7	51.5	0.673
PCA	1st visit	66.1	70.0	64.8	0.691
	1st + 2nd visit	57.1	55.6	60.0	0.625
RBF	1st visit	66.1	69.3	71.4	0.705
	1st + 2nd visit	70.1	74.2	65.7	0.754
RF	1st visit	73.2	75.6	77.2	0.788
	1st + 2nd visit	75.0	74.1	76.1	0.796

**(d):** 

Method	Timepoints	ACC	SEN	SPE	AUC
SVM	1st visit	65.0	64.6	67.2	0.698
	1st + 2nd visit	70.0	68.3	73.4	0.756
PCA	1st visit	53.0	55.7	59.1	0.578
	1st + 2nd visit	53.0	55.6	60.2	0.577
RBF	1st visit	62.3	64.3	66.5	0.684
	1st + 2nd visit	67.6	70.1	70.7	0.788
RF	1st visit	65.2	69.0	63.2	0.724
	1st + 2nd visit	65.2	69.2	63.9	0.727

**(a):** 

Method	Timepoints	ACC	SEN	SPE	AUC
SVM	1st visit	83.3	81.4	87.9	0.843
	1st + 2nd visit	89.2	83.2	92.4	0.934
PCA	1st visit	75.0	74.7	78.1	0.796
	1st + 2nd visit	76.7	75.2	79.9	0.814
RBF	1st visit	68.3	66.5	75.3	0.756
	1st + 2nd visit	78.3	80.9	70.4	0.852
RF	1st visit	85.0	88.4	79.7	0.911
	1st + 2nd visit	87.7	90.2	81.4	0.924

**(b):** 

Method	Timepoints	ACC	SEN	SPE	AUC
SVM	1st visit	80.0	84.5	78.9	0.857
	1st + 2nd visit	82.0	86.1	79.7	0.871
PCA	1st visit	68.3	65.2	70.0	0.743
	1st + 2nd visit	78.8	80.7	68.4	0.808
RBF	1st visit	62.7	64.1	59.3	0.676
	1st + 2nd visit	89.2	91.8	86.2	0.934
RF	1st visit	80.4	84.2	77.6	0.855
	1st + 2nd visit	82.4	86.3	79.5	0.876

**(c):** 

Method	Timepoints	ACC	SEN	SPE	AUC
SVM	1st visit	66.0	68.3	59.9	0.713
	1st + 2nd visit	64.7	69.4	61.3	0.698
PCA	1st visit	64.0	67.0	61.2	0.692
	1st + 2nd visit	65.0	68.1	62.0	0.708
RBF	1st visit	67.9	70.4	66.4	0.735
	1st + 2nd visit	66.0	69.3	64.2	0.739
RF	1st visit	82.1	85.6	80.1	0.875
	1st + 2nd visit	81.7	84.5	79.1	0.888

**(d):** 

Method	Timepoints	ACC	SEN	SPE	AUC
SVM	1st visit	51.7	54.5	57.8	0.594
	1st + 2nd visit	55.0	58.2	63.9	0.643
PCA	1st visit	60.9	65.6	59.1	0.663
	1st + 2nd visit	65.7	70.8	64.0	0.712
RBF	1st visit	65.0	68.1	62.5	0.704
	1st + 2nd visit	68.2	71.5	65.7	0.801
RF	1st visit	72.5	79.0	68.7	0.785
	1st + 2nd visit	72.5	79.2	69.9	0.790

**Table 5:** Comparison in classification accuracy between FDG-PET and AV45-PET using combination of baseline and second-visit timepoints for all diagnostic pairs.

	FDG-PET results			AV45-PET results		
	RBF-SVM	Random forest	*P* value	RBF-SVM	Random forest	*P* value
AD versus NC	91.7%	91.2%	0.010	90.8%	87.9%	0.024
AD versus EMCI	85.7%	85.7%	0.015	80.0%	88.0%	0.024
AD versus LMCI	87.5%	79.2%	0.016	88.9%	81.5%	0.027
EMCI versus NC	63.3%	56.7%	<0.01	57.7%	59.7%	0.0203
LMCI versus NC	63.5%	65.4%	0.018	61.2%	55.7%	<0.01
EMCI versus LMCI	53.9%	64.1%	<0.01	52.2%	56.5%	0.017

## Data Availability

Data used in preparation of this article were obtained from the Alzheimer's Disease Neuroimaging Initiative (ADNI) database (http://adni.loni.usc.edu). As such, the investigators within the ADNI contributed to the design and implementation of ADNI and/or provided data but did not participate in analysis or writing of this report. A complete listing of ADNI investigators can be found at http://adni.loni.usc.edu/wp-content/uploads/how_to_apply/ADNI_Acknowledgement_List.pd.
